# Ultrafine and High-Strength Silk Fibers Secreted by Bimolter Silkworms

**DOI:** 10.3390/polym12112537

**Published:** 2020-10-30

**Authors:** Kaiyu Guo, Xiaolu Zhang, Zhaoming Dong, Yuhui Ni, Yuqing Chen, Yan Zhang, Haoyun Li, Qingyou Xia, Ping Zhao

**Affiliations:** 1 State Key Laboratory of Silkworm Genome Biology, Southwest University, Chongqing 400716, China; kaiyug@163.com (K.G.); xiaolushg@126.com (X.Z.); niyu199901@email.swu.edu.cn (Y.N.); cyq13042326572@163.com (Y.C.); lihaoyunswu@126.com (H.L.); 2 Biological Science Research Center Southwest University, Chongqing 400716, China; dong-zhaoming@163.com (Z.D.); zhangy66@swu.edu.cn (Y.Z.); xiaqy@swu.edu.cn (Q.X.); 3 Chongqing Engineering and Technology Research Center for Novel Silk Materials, Chongqing 400716, China

**Keywords:** ultrafine silk fibers, bimolter, silkworm, mechanical properties

## Abstract

Ultrafine fibers are widely employed because of their lightness, softness, and warmth retention. Although silkworm silk is one of the most applied natural silks, it is coarse and difficult to transform into ultrafine fibers. Thus, to obtain ultrafine high-performance silk fibers, we employed anti-juvenile hormones in this study to induce bimolter silkworms. We found that the bimolter cocoons were composed of densely packed thin fibers and small apertures, wherein the silk diameter was 54.9% less than that of trimolter silk. Further analysis revealed that the bimolter silk was cleaner and lighter than the control silk. In addition, it was stronger (739 MPa versus 497 MPa) and more stiffness (i.e., a higher Young’s modulus) than the trimolter silk. FTIR and X-ray diffraction results revealed that the excellent mechanical properties of bimolter silk can be attributed to the higher β-sheet content and crystallinity. Chitin staining of the anterior silk gland suggested that the lumen is narrower in bimolters, which may lead to the formation of greater numbers of β-sheet structures in the silk. Therefore, this study reveals the relationship between the structures and mechanical properties of bimolter silk and provides a valuable reference for producing high-strength and ultrafine silk fibers.

## 1. Introduction

*Bombyx mori* silk fiber has a long history of application in the textile industry since it was discovered; the fibers were unwound from cocoons and used to manufacture strong and unique fabrics. However, the rapid development of modern fiber technologies has led to stricter requirements for the fineness of such silk fibers. In recent years, ultrafine fiber has been widely used in many fields because of its excellent characteristics, including its high detergency, heat preservation, softness, and lightness [1,2,3]. Ultrafine fiber can be converted into ultrafine fabric, where the fabric surface is extremely smooth, and it exhibits minimal air resistance; its optical properties (e.g., the refractive index, transmittance, and reflectance) differ significantly than traditional fabric. Indeed, the light weight fabrics and clean cloths composed of ultrafine fibers are popular in Japan and Europe.

In sericulture, thin silk fibers are typically obtained by breeding trimolter strains or inducing normal tetramolters into trimolters. This process is followed since trimolter silk is thinner than typical tetramolter silk, which suggests that further reducing the number of molts could produce thinner silk. To date, numerous studies have focused on trimolters induced by hormone-like chemicals [4,5,6]. For example, Asano et al. first introduced and successfully applied anti-juvenile hormones (AJHs) that induce tetramolter silkworms into trimolters [4], whereas Zhuang et al. induced two Chinese tetramolter silkworm species into trimolters using the imidazole compound YA_20_ [5]. In addition, Niu et al. obtained trimolter silkworms and found that the obtained trimolter silk exhibited thin filaments, high reliability, excellent neatness, and little filament deviation [6]. In the textile industry, linear density is generally used as a specification corresponding to silk fineness. More specifically, ultrafine fiber is defined as a filament with a fineness of less than 1 dtex [7]; thus, the fineness of trimolter silk (i.e., >1.5 dtex) does not meet modern expectations. Therefore, the induction of bimolters to produce finer silk fibers has been considered. Unfortunately, normal tetramolter silkworms cannot be induced into bimolters, which have been previously induced from trimolter strains [8]. Under this previously reported treatment, trimolter larvae could be induced into bimolter which spun cocoons comprising ultrafine silk. However, the study of bimolter silk is an essentially undeveloped field, and the structure and properties of bimolter silk remain unknown.

In our previous study, we induced trimolters to obtain thinner silk fibers compared to those of the corresponding tetramolters. Interestingly, we found that the conformation of trimolter silk fibroin possessed a higher content of β-sheet structures and superior mechanical properties compared to the normal tetramolter silk fibroin [9]. A number of studies have also reported that the mechanical properties of silk fibers are closely related to their diameter [10,11,12], where thin silk fiber has a higher breaking strength than that coarse fiber [10,11]. Evidently, thin fibers with small filaments are strong because of their high hydrogen bond energy density [12], and this can result in the formation of thinner silk fibers and improved silk performances. In general, silkworm silk is considered significantly weaker than spider dragline silk [13]. To date, various physical, chemical, and biological approaches have been proposed and implemented to enhance the mechanical properties of silkworm silk [14,15,16,17,18]. In this context, post-functionalization approaches have most often been applied to produce high-strength silk fibers from regenerated silk fibroin solutions containing additives through dry or wet spinning [19,20]. However, these approaches inevitably require the use of toxic solvents and complex multistep procedures.

Thus, we report on the treatment of a trimolter silkworm strain with the AJH KK-42 to induce bimolters and obtain ultrafine silk fibers. In addition, the strength and Young’s modulus of the filament silk of the bimolter are determined. Furthermore, the morphologies of the bimolter cocoon and silk are observed by scanning electron microscopy (SEM), and the crystalline structure of the silk is characterized and analyzed via attenuated total internal reflection Fourier transform infrared spectroscopy (ATR-FTIR) and X-ray diffraction (XRD) measurements. Finally, the silk gland structure of the bimolter is also used to explain the improved mechanical properties of the obtained silk.

## 2. Materials and Methods

### 2.1. AJH Induction of Silkworm Bimolters

The trimolter mutant strain named *Qiang zhongxian* was supplied by Professor Ping Chen (College of Biotechnology, Southwest University, China). Generally, silkworms undergo four larval molts and form five larval instars; however, for the purpose of this study, the trimolter mutant with a characteristic of three molting times was employed. All trimolter larvae were reared on fresh mulberry leaves at a constant temperature of 25 °C on a 12:12 h light:dark cycle. We collected individual larvae on day 1 of the third instar stage for AJH treatment. There are fifteen trimolters in each group. The AJH KK-42 (1-benzyl-5[(E)-2,6-dimethyl-1,5-heptadienyl]) (SIMR Biotech, Shanghai, China) was dissolved in acetone to a concentration of 0.5 mg/mL. In the treatment group, doses of 0.5 μg KK-42 per larva were applied on day 1 of the third instar stage larvae along the dorsal midline of the larval thorax. As a control, trimolter larvae treated with the same dose of acetone were used. The KK-42 and acetone solution were applied on treatment and control groups only once, respectively.

### 2.2. Chitin Staining of Silk Glands

The silk glands were fixed overnight in 2.5% glutaraldehyde and washed three times for 5 min with phosphate-buffered saline (PBS, pH 7.2); then, the sample was further fixed in 30% sucrose for 8 h. The anterior silk gland was cut for tissue embedding in the optical cutting temperature freezing agent (Sakura, San Mateo, CA, USA). Subsequently, the embedded fragments were cross-sectioned into 8 μm slices at −30 °C using a Leica CM1950 microtome (Leica Microsystems, Frankfurt, Germany) and attached to a glass slide. After washing three times (each for 10 min) in PBS, the slices were incubated with DAPI staining solution (Beyotime, Shanghai, China) for 10 min and washed three times with phosphate-buffered saline containing Tween 20 (PBST). Finally, the slices were incubated with fluorescein isothiocyanate (FITC)-labeled wheat germ agglutinin (WGA) (Sigma, Saint Louis, MS, USA) and washed with PBST. The slices were observed under a microscope (Olympus BX51, Hatagaya, Japan).

### 2.3. Morphology Observations

The surface and cross-sectional morphologies of the silk and the cocoon were observed using a Leica DM1000 microscope and by SEM (JSM-6510LV, Shoshima, Japan) with an accelerating voltage of 15 kV and magnifications of 100–3000×. The silk samples were soaked in Coomassie brilliant blue R250 dye for 15 min prior to obtaining the light microscope observations. The diameter of the cocoon silk fiber (each containing two brin filaments) was measured according to a previous literature report [21].

### 2.4. Filament Size Testing

The raw silk size testing method was employed using the tex count system [22]. The cocoon was placed into a boiling NaHCO_3_ solution (0.5% *w*/*v*) for 2 min, and then, the single silk fiber was reeled using a gauge reel. During this process, a single silk fiber was simultaneously obtained and degummed. All samples were dried in the oven before weighting. The length of the single silk fiber was obtained from the gauge reel; the dtex value is the fineness unit, which refers to the weight of 10,000 m of silk, i.e., 1 dtex = 1 g/10,000 m. In our case, 10 m of silk fiber was weighed and multiplied by 1000.

### 2.5. Cleanliness Test of the Silk

Cocoon silk samples of the same weight were taken from the treatment and control groups and placed in 50 mL centrifuge tubes with equal part of distilled water. All samples were placed on a shaking table with a speed of 250 rpm at 25 °C for 60 min. Then, the silk and the impurities were filtered through a fine sieve, and the filtrate was poured into clean centrifugal tubes. A turbidimeter (Lamotte, Chestertown, MD, USA) was used to test the cleanliness of the silk samples. Each test required 10 mL of filtered liquid. Five biological repetitions were carried out.

### 2.6. ATR-FTIR Analysis

Cocoons from bimolters and trimolters were chosen randomly and degummed as described previously [23]. The silk samples were then washed and air-dried at room temperature. IR spectroscopy was performed in the ATR mode using a Thermo Scientific Nicolet iN10 with a slide-on ATR objective lens. The spectra were collected in the range (650–4000 cm^−1^) at a resolution of 8 cm^−1^ with 256 coadded scans [24], and an applied ATR pressure of 75 psi. OMNIC 9 software (Thermo Scientific) was used to collect and process the spectral data. The background was collected before collecting the ATR spectra of the samples. Spectral data analysis, including baseline correction, deconvolution of the amide I bands, and peak fitting, was performed using OMNIC 9 software (Thermo Scientific) and PeakFit software (Seasolve, version 4.12) [25,26,27]. Each spectrum represents the mean of separate deconvolutions for at least 15 separate samples.

### 2.7. XRD Analysis

XRD was used to identify the crystalline phases present in the micro-samples. All XRD measurements were obtained using an X’Pert^3^ Powder X-ray diffractometer (PANalytical, Almelo, Netherlands) with Cu-Kα radiation from a source operated at 40 kV and 40 mA. The silk samples were mounted on aluminum frames and scanned from 5° to 50° (2θ) at a scan rate of 2.0°/min. The relative crystallinity of each sample was calculated using MDI JADE 6.5 software. During the deconvolution process, the numbers and positions of the peaks were fixed using previously reported methods [28,29,30]. The sample crystallinity was evaluated according to the following Equation (1):

Crystallinity = (X/Y) × 100%(1)

where X is the net area of the crystal peaks, and Y is the net area of crystal peaks + amorphous halo [31,32,33].

### 2.8. Mechanical Testing

Mechanical testing was conducted using single silk fiber spun by the bimolters and the control trimolters. The cocoons were harvested and degummed as described in Section 2.6. The degummed silks were used as samples for characterization of the mechanical properties of the silk fibers. In this case, two fibroin brins were completely separated [23,25,34]. A single brin was used to test the mechanical properties. To ensure the accuracy of the data, we also used the remainder of the same brin for SEM to calculate the diameter for mechanical testing. A minimum of 25 tensile deformation tests were performed for each silk type on fibers from five separate cocoons. The tensile properties of individual brins were measured using a DMA-Q800 dynamic mechanical analyzer (TA, New Castle, DE, USA). Mechanical tests were performed according to a previously reported method [9]. For each tensile test, the fiber diameter was measured using JCM-5000 SEM (JEOL, Shoshima, Japan). The experimental data obtained relating to the mechanical properties were analyzed using the TA Universal Analysis software. Subsequently, the raw data were used to calculate the mechanical performance parameters using the ORIGIN8.0 software (OriginLab, Northampton, MA, USA).

## 3. Results

### 3.1. KK-42-Induced Bimolters and Changes in the Cocoon

**Figure 1:**
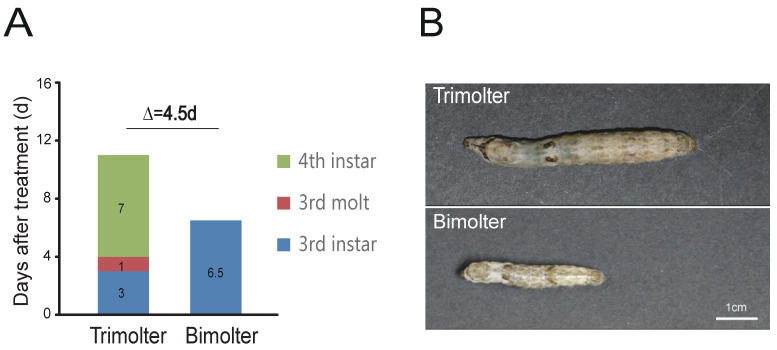
Schematic diagram of the developmental progression difference after separately applying KK-42 and acetone. (**A**) The bimolters were trimolters treated with KK-42 on day 1 of the third instar, and the control group were trimolters treated with the same volume of acetone on day 1 of the third instar. (**B**) Morphologies of the trimolter and bimolter before wandering. Bars represent 1 cm.

**Figure 2:**
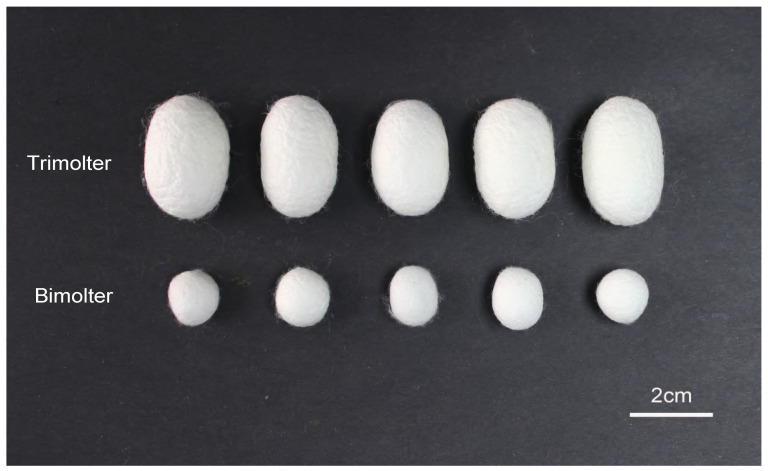
Photos of cocoons from the trimolter and bimolter. Bars represent 2 cm.

Normal tetramolter silkworms undergo four larval molts and cannot be directly induced to bimolters. In this study, a trimolter silkworm strain was instead used to induce bimolters, where the AJH KK-42 was used to treat the trimolter mutant strain on day 1 of the third instar stage. After KK-42 treatment, 92% of the trimolters were successfully induced into bimolters. The mortality rate of the bimolter silkworm after the anti-juvenile hormone treatment was <1%. The third instar stage of bimolter larval increased from 3 to 6.5 d, but no fourth instar was observed, in contrast to the case of the control trimolter sample. Finally, the larvae underwent two larval molts, and the larval stage was shortened by 4.5 d (Figure 1A). Owing to developmental changes, the bodies of the bimolters before spinning were significantly smaller than those of the control trimolters (Figure 1B), and their cocoons had smaller volumes (Figure 2). According to the weight data (Supplementary Figure S1), the cocoon shell weight of the treatment groups was 85% lower than that of the control groups (*p* < 0.001). However, the cocoon shell percentages of the bimolters and trimolters did not differ significantly.

### 3.2. Morphological Characteristics of the Cocoon and Silk

**Figure 3:**
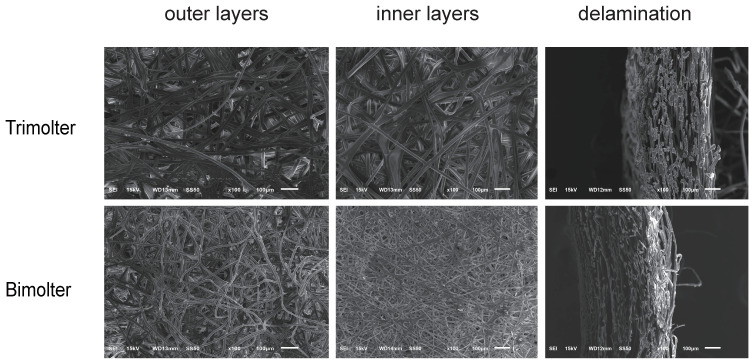
SEM micrographs of the outer layers, inner layers and delamination of cocoons obtained from bimolter treatment groups and trimolter control groups. Bars represent 100 μm.

**Figure 4:**
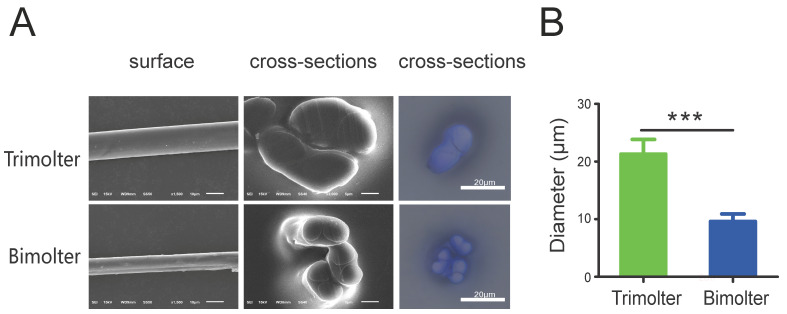
Silk morphologies and diameter. Cocoon silk from the trimolter applied KK-42 and acetone separately on the day-1 of 3rd. (**A**) Surface and cross-sections morphologies of silk were photographed by SEM and optical microscope. Bars represent 10 μm and 5 μm respectively. (**B**) The averaged diameter of trimolter and bimolter silk fibers, *n* = 25. Bars represent 10 μm. *** represents *p* < 0.001 (Student’s *t*-test). Error bars, SD.

The bimolter cocoon exhibited a relatively high packing density, with very densely packed thin fibers and thus small apertures. In contrast, the cocoons of the trimolter control groups were loosely packed with thick silk fibers and large apertures (Figure 3). According to the cocoon delamination images, the bimolter cocoon was thinner than that of the trimolter (Figure 3). SEM and light microscopy images of the cocoon cross-sections and surfaces illustrate their detailed fiber dimensions and morphologies (Figure 4A). All silk fibers consisted of two fibroin brins surrounded by sericin; the fiber morphology appeared unchanged after treatment, but the diameter was significantly different. The bimolter silk fiber clearly had a smaller diameter than that of the trimolter silk, as confirmed by cross-sectional SEM and light microscopy images. The diameters of the two silks were measured using SEM, and the average diameter of the bimolter silk fibers decreased from that of the trimolter silk, i.e., from 21.3 ± 0.51 μm (mean ± standard deviation, SD) to 9.60 ± 0.26 μm (Figure 4B). In addition, the fineness of the bimolter silk was only 0.6–0.8 dtex, which meets the standard for ultrafine fiber.

### 3.3. Quality Characteristics of Silk

The lightness and cleanliness of the silk were investigated. The silk was weighed in 500 m lengths and the lightness of each sample compared. The average weight of the bimolter silk decreased from 0.0745 ± 0.001 g (mean ± SD, i.e., the weight of the trimolter silk) to 0.03775 ± 0.0005 g, which is a reduction of 49.3%. A turbidimeter was used to test the cleanliness of the silk. More specifically, after subjecting the sample to shaker rocking for 60 min, we examined the turbidity of the silk in an aqueous solution. The average turbidity of the bimolter silk aqueous solution was reduced from that of the trimolter silk, i.e., from 4.494 ± 0.0752 NTU to 3.968 ± 0.0635 NTU (*p* < 0.001). Because lower turbidity leads to better cleanliness, our result indicated that the ultrafine silk fiber exhibited good cleanliness to the control silk.

### 3.4. Chitin Staining of the Silk Glands

**Figure 5:**
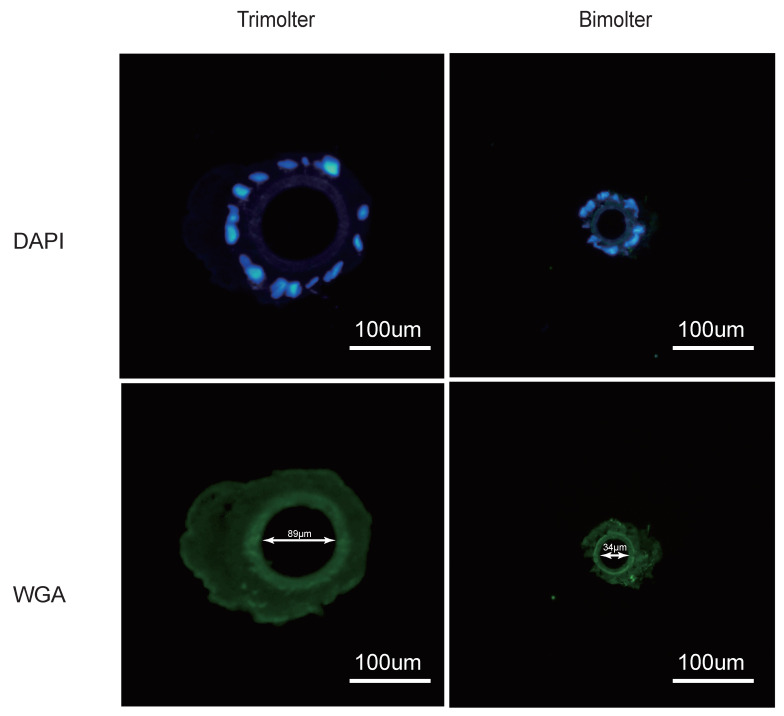
Chitin-stained of anterior silk gland from bimolter treatment groups and trimolter control groups. Slides were stained with wheat germ agglutinin (WGA) (green), whereas the nucleus was stained with DAPI (blue). The innermost green ring is the silk gland endometrium consisted of the chitin layer.

The bodies and anterior silk glands of the bimolters before spinning were significantly smaller than those of the trimolters (Figure 1B), suggesting that changes in the growth process affected both tissue and organ development. It should be noted here that the silk gland is the only organ where silk proteins are synthesized and secreted in the silkworm, and previous studies have indicated that the secondary structure of silk proteins mainly changes in the anterior silk gland [35,36]. To investigate the cause of the thinner silk fibers after bimolter induction, the chitin layer of the anterior silk gland was stained with lectin and DAPI dihydrochloride solution (Figure 5). The relative size of the lumen (surrounded by the chitin layer) appeared to vary in tandem with the size of the silkworm body. The diameter of the bimolter lumen decreased from 89 to 34 μm, compared with that of the trimolter lumen (i.e., diameter of 89 μm). Therefore, our analysis suggests that the anterior silk gland lumen in bimolters is considerably smaller than that in trimolters.

### 3.5. Effect of Bimolter Induction on the Secondary Structure of Silk Fibers

**Figure 6:**
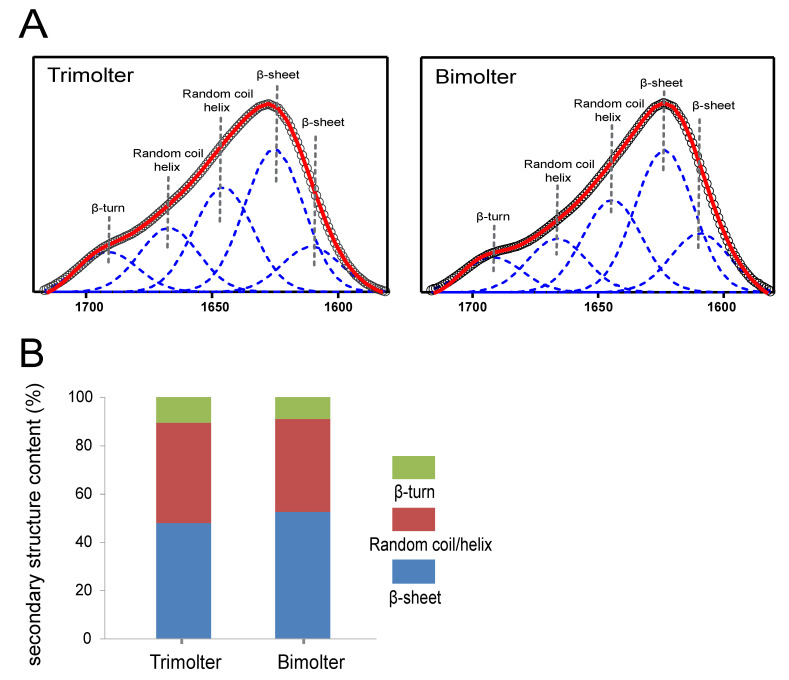
Attenuated total internal reflection Fourier transform infrared spectroscopy (ATR-FTIR) spectra of silk fibers. (**A**) Deconvolution results of the amide I band of trimolter and bimolter silk fibers. (**B**) Comparison of the secondary structures of the silk fibers from different group; circles, original spectrum; dashed curve, deconvoluted peaks; red curve, simulated spectrum from summed peaks.

The conventional FTIR technique is unsuitable for testing fine silk fibers because the spot size of the conventional Globar light source (usually a minimum of 10 μm × 10 μm) is larger than the average diameter of the bimolter silk fiber (<10 μm, Figure 4B). Therefore, only a small part of the infrared beam can illuminate a single silk filament, which results in a poor-quality image and an essentially useless spectrum. Many studies have shown that the quality of the ATR-FTIR spectra of silk fibroin is comparable to that of ordinary FTIR spectra [27,31,37]. Therefore, we used ATR-FTIR to characterize the silk secondary structures because it is not limited by the sample diameter or size [38,39,40]. The amide I spectral region is commonly used for the analysis of secondary protein structures [41,42,43], wherein the amide I bands at 1624 and 1620 cm^−1^ usually correspond to β-sheet structures [26,35,44,45], the bands between 1688 and 1666 cm^−1^ correspond to β-turn structures [46], and those between 1665 and 1654 cm^−1^ correspond to helix structures [26,44]. The results presented in Figure 6A clearly revealed that the secondary silk fiber structures of the trimolters and bimolters differed from one another. The compositions of the secondary structures present in trimolter silk were as follows: 47.8 ± 0.356% β-sheet, 41.6 ± 0.295% random coil or helix, and 10.6 ± 0.115% β-turn (Figure 6B). However, following KK-42 treatment, these compositions became 52.5 ± 0.289% β-sheet, 38.5 ± 0.204% random coil or helix, and 9.0 ± 0.095% β-turn (Figure 6B). These results indicate that greater quantities of the β-sheet conformation were present in the bimolter silk (i.e., 52.5 ± 0.289% cf. 47.8 ± 0.356 % for trimolter silk). In contrast, the random coil and helical contents decreased from 41.6 ± 0.295 to 38.5 ± 0.204% after KK-42 treatment.

### 3.6. Crystalline Structure Characteristics of Silk Fibers

**Figure 7:**
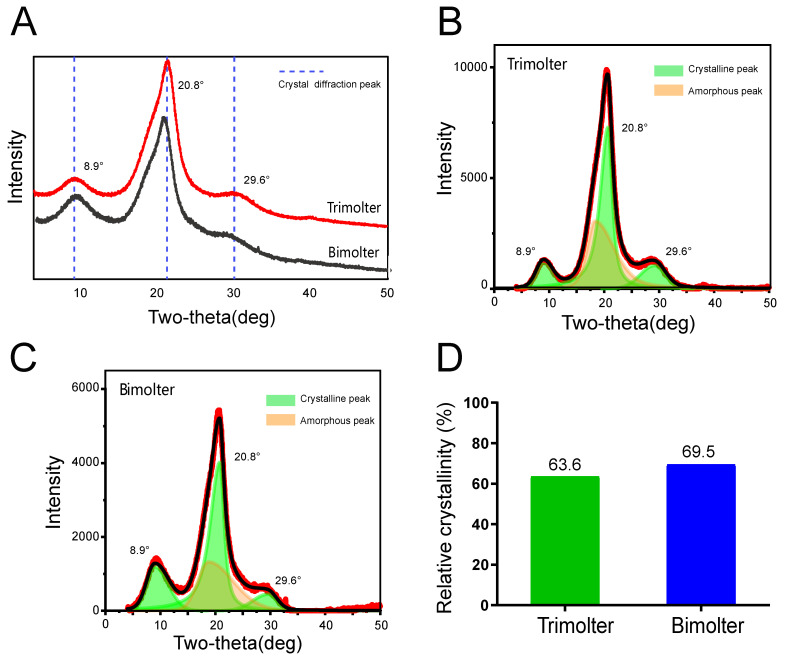
(**A**) X-ray diffraction diffractograms of trimolter and bimolter silk. (**B**) Deconvolution of the trimolter silk diffractogram. (**C**) Deconvolution of the bimolter silk diffractogram. The peaks are fitted as the sum of five Gaussians: Three crystalline peaks (light green) and one amorphous halos (pale orange). The three crystalline peaks are indexed as 8.9°, 20.8 and 29.6°. (**D**) Relative crystallinity of trimolter and bimolter silk.

The crystalline structures of the silk fibers were confirmed by XRD measurements. Previous research has shown that the XRD patterns of protein fiber materials can be categorized; the peaks at 11.95, 21.4, and 24.02° correspond to the α-helix crystal structure, and those at 9.1, 20.6, 24.6, 29.3, and 30.90° correspond to the β-sheet crystal structure [28,29,47,48]. In the present study, crystal diffraction peaks were detected at approximately 8.9, 20.8, and 29.6° for the trimolter and bimolter silk samples (Figure 7A). The deconvolutions of the different silk diffractograms are shown in Figure 7B,C; it is apparent that the crystal diffraction peaks differed, thereby supporting the ATR-FTIR result (Figure 6). Crystallinity analysis showed that the crystallinity of the silk increased from 63.6% for the trimolter sample to 69.5% for the bimolter sample (Figure 7D).

### 3.7. Mechanical Properties of the Silk Fiber

**Figure 8:**
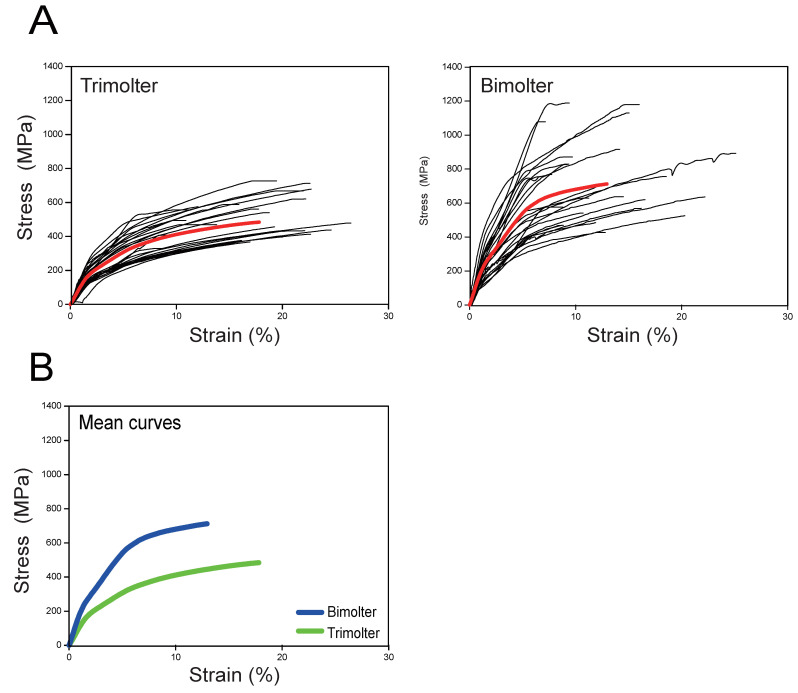
Tensile stress–strain curves. (**A**) Stress–strain curves of silk fibers from each group (*n* = 25). The bold red curve represents the mean curve from each group. (**B**) Comparison of the mean curves of the trimolter silk (green) and bimolter silk (blue).

**Figure 9:**
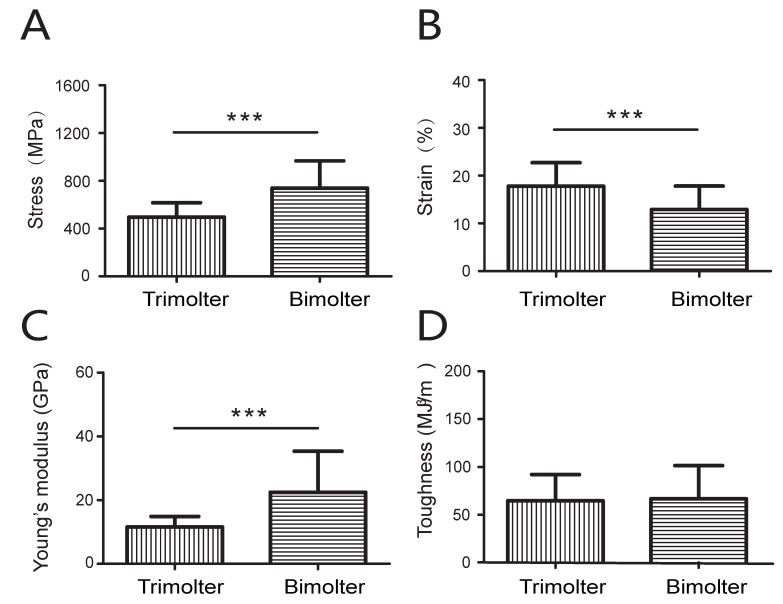
Mechanical parameters of silk fibers from trimolter and bimolter, *n* = 25. (**A**) Maximum stress. (**B**) Maximum strain. (**C**) Young’s modulus. (**D**) Toughness. *** represent *p* < 0.05 and *p* < 0.001, respectively (Student’s *t*-test). Error bars, SD.

**Figure 10:**
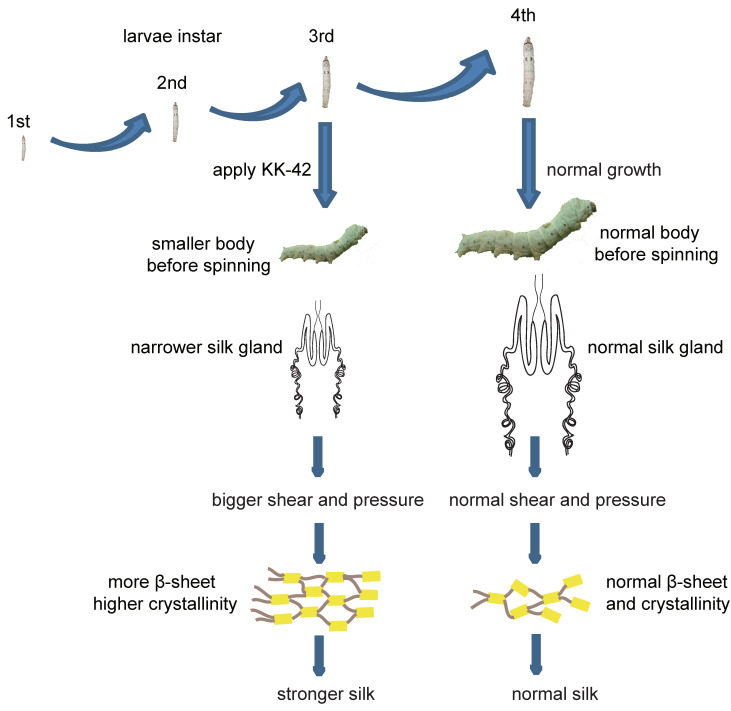
Model of the improvement in the mechanical properties of silk induced by anti-juvenile hormone (AJH).

Degummed silk from cocoons was used to determine the mechanical properties of the silk fibers. Analysis of the stress–strain curves indicated that the bimolter silk was stronger than the trimolter silk (Figure 8). Figure 9 showed the mechanical parameters, including the maximum strength, elongation, Young’s modulus, and toughness of single trimolter and bimolter silk brin. More specifically, the trimolter and bimolter silk samples exhibited a maximum strength of 496 and 739 MPa, respectively (*p* < 0.001; Figure 9A), and Young’s moduli of 11.6 and 22.5 GPa, respectively (*p* < 0.001; Figure 9C). In addition, the bimolter silk reached an elongation of 12.9%, which was significantly lower than that of 17.8% of the trimolter silk (*p* < 0.001; Figure 9B). However, the toughness values of the trimolter and bimolter silk samples did not differ significantly (Figure 9D). These results suggested that the silk fibers from the KK-42-induced bimolters had improved in strength and stiffness with respect to those from the control trimolters. A dynamic model for the improved mechanical properties of silk is presented in Figure 10. More specifically, the normal growth and development of trimolters were altered by the addition of KK-42, resulting in a smaller silk gland. When the silk protein flowed into the narrow silk gland, it was subjected to larger shearing force and pressure; this caused the structure of the silk fibers to change. Finally, higher crystallinity and a greater number of β-sheets increased the ultimate strength and Young’s modulus of the silk.

## 4. Discussion

Ultrafine silk has particular economic value and significant potential for application in a range of fields, and as a result, methods of inducing silkworm larvae to spin thin silk have attracted significant research interest. Silkworms spin silk throughout their larval stages; the diameters of the spun fibers vary among larval instars. In general, the smaller the body of the silkworm, the thinner the silk. In this study, an appropriate dose of KK-42 was used topically to the third instar trimolter larvae to induce precocious metamorphosis. This change during growth also affected tissue and organ development, with the body sizes and silk glands of the bimolters differing significantly, relative to those of the control trimolters.

Typically, silkworms undergo four molts (i.e., tetramolters) and spin cocoons during the fifth larval instar. The growth and molting of silkworms are tightly regulated by two hormones: Ecdysone, which causes molting, and juvenile hormone (JH), which prevents metamorphosis [49,50]. Breaking this hormone balance alters the growth and molting processes, which can convert normal silkworms into trimolters or pentamolters, which exhibit one fewer or one more instar stage, respectively [51,52]. The compounds that induce precocious metamorphosis or other symptoms of JH deficiency in insects are collectively called AJH agents, and these function by interfering with the biosynthesis, transport, or secretion of hormones [53,54]. Several AJH agents have been used to induce trimolters for producing thin silk fibers such as the JH inhibitors SD-III, SM-1, and YA_20_, [5,55,56,57]. Although these methods cause the silk spun by trimolters to become thinner, the diameter remains too wide to be considered ultrafine fiber. Nonetheless, this tendency suggests that silkworms can be further treated to spin even thinner silk. Additionally, our previous studies have shown that the mechanical properties of the silk of trimolters induced by KK-42 are better than those of the tetramolter silk. Thus, to obtain thinner and stronger silk, we attempted to induce normal tetramolter silkworms into bimolters; however, this was unsuccessful. Therefore, in this work, we applied KK-42 to a trimolter mutant strain and successfully induced bimolters. The average diameter of the resulting bimolter silk was less than 10 μm.

Compared with that of the trimolter mutant, the shortened period of larval development resulted in a notably smaller body (Figure 1B), which may affect the silk gland. Previous studies have shown that the liquid silk fibroin flows from the posterior silk gland to the anterior silk gland, during which the conformation of the silk fibroin changes gradually from a helical structure to a β-sheet structure [35,58]. Analysis of chitin-stained silk glands also indicated that the anterior silk gland was significantly smaller in bimolter larvae (Figure 5). These small anterior silk glands lead to the production of silk fibers with smaller diameters as well as different structures. Furthermore, they induce a greater compressive stress and shearing force on the silk protein solution in the anterior silk gland, which may result in a more compact arrangement of fibroin molecules within the silk [59,60]. Recent studies have indicated that the shearing force plays a key role in the transition of the silk protein conformation from a random coil and/or helical conformations to β-sheets in the anterior silk gland [60,61,62]. The structural and size changes of the anterior silk glands observed in this study were therefore expected to provide greater shear and compressive stresses in the anterior silk gland to result in the formation of additional β-sheet structures and give a higher crystallinity in the silk protein. In general, increases in the number of β-sheets and crystallinity are associated with an increase in rigid connections between molecules in the silk fiber. This increases the strength and elastic modulus of the silk fiber because β-sheets are responsible for the physical properties and highly dense crystal structure [31,63,64,65,66,67]. We also found that the extensibility of the silk fibers decreased after KK-42 treatment, which may be owing to a relatively low content of random coil and β-turn structures in the bimolter silk. In addition, it has been reported that thinner silk has higher strength and stiffness because of its high hydrogen bond energy density [12]; thinner silk also exhibited a higher “draw ratio” when a polymer jet was applied to a stenotic lumen [11]. Notably, it was reported that the improved strength was not only originated from the increase in crystallinity but may due to the improved degree of orientation or alignment of the silk molecular chains [68]. We considered it was possible. The work will be investigated in the following study.

The demand for silk products is developing towards diversification and functionalization. Ultrafine silk fiber has the advantages of lightness, strength, and cleanliness, and it has been used to produce high-grade and special-purpose silk products for textiles [6,69]. The ultrafine fibers reported previously were mainly prepared via a composite spinning technique, which requires complex technologies and incurs high costs [7]. In contrast, the method employed in this study is a safe, simple and an efficient approach to obtain high-strength and ultrafine silk fibers directly from silkworms. In addition, the growth cycle of bimolters in sericulture was shortened, which will not only save labor time and decrease costs but also reduce the occurrence of silkworm diseases. Moreover, the greater economic value and potential advantages of bimolter silk indicate that it will have a bright future.

## 5. Conclusions

We reported on the treatment of a trimolter mutant silkworm strain with the anti-juvenile hormone (AJH) KK-42 to induce bimolters, which produced more closely packed cocoons and ultrafine silk fibers. In addition, attenuated total internal reflection Fourier transform infrared spectroscopy (ATR-FTIR) showed that the bimolter silk fibroin possessed a higher content of β-sheet structures and fewer random coil and helical structures. The X-ray diffraction measurements revealed that the bimolter silk exhibited a more crystalline structure than the control trimolter silk, whereas mechanical testing indicated that the bimolter silk fibers were stronger and stiffer. We also stained the silk glands with chitin to investigate the origin of the enhanced mechanical properties of the silk sample, and it was found that the anterior silk gland lumen in bimolters is significantly smaller than that in trimolters. In summary, this study reveals the relationship between the morphological changes of bimolter silkworms and the structures and mechanical properties of the resulting silk fibers, thereby providing a valuable fundamental reference for the production of ultrafine and high-strength silk fibers.

## Supplementary Materials

The following are available online at https://www.mdpi.com/2073-4360/12/11/2537/s1, Figure S1.

## Abbreviations

AJH: anti-juvenile hormone; FTIR: Fourier-transform infrared spectroscopy; ASG: anterior silk gland; SEM: scanning electron microscope; WGA: wheat germ agglutinin-FITC labeled; XRD: X-ray diffraction; ATR: attenuated total reflectance; DMA: dynamic mechanical analyzer; PBS: Phosphate buffered saline.
