# Supplementary figures and images for: Ultrafine and High-Strength Silk Fibers Secreted by Bimolter Silkworms
Source: Polymers (Basel). 2020 Oct 30;12(11):2537. doi: 10.3390/polym12112537 (PMC7693878; doi:10.3390/polym12112537)

# Supplementary Figure S1

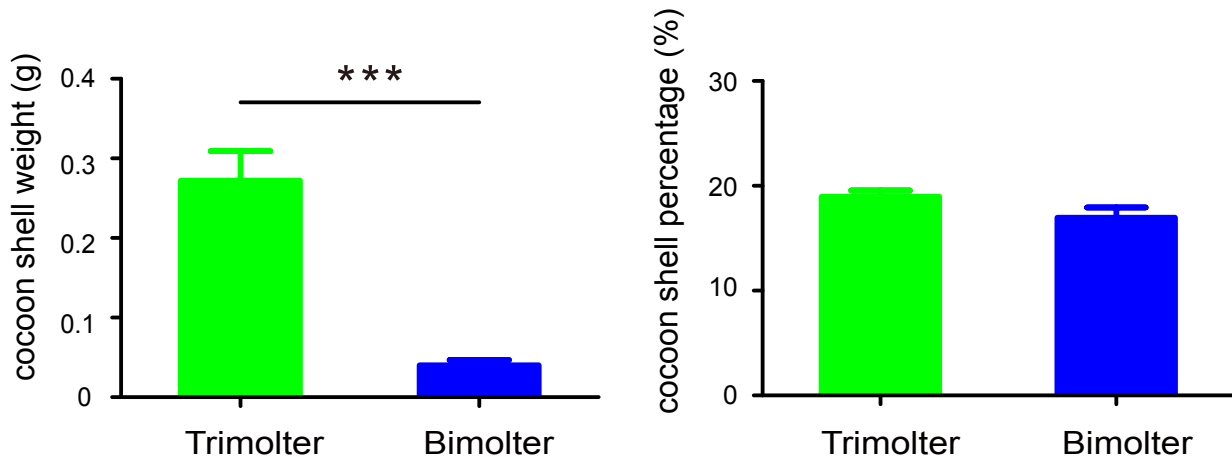

Supplement: Supplementary file 1 [file polymers-12-02537-s001.pdf]
